# Heterogeneity in District-Level Transmission of Ebola Virus Disease during the 2013-2015 Epidemic in West Africa

**DOI:** 10.1371/journal.pntd.0004867

**Published:** 2016-07-19

**Authors:** Fabienne Krauer, Sandro Gsteiger, Nicola Low, Christian H. Hansen, Christian L. Althaus

**Affiliations:** 1 Institute of Social and Preventive Medicine (ISPM), University of Bern, Bern, Switzerland; 2 MRC Tropical Epidemiology Group, London School of Hygiene & Tropical Medicine, London, United Kingdom; Mwanza Intervention Trials Unit, National Institute for Medical Research, Mwanza, Tanzania; University of Liverpool, UNITED KINGDOM

## Abstract

The Ebola virus disease (EVD) epidemic in West Africa in 2013–2015 spread heterogeneously across the three hardest-hit countries Guinea, Liberia and Sierra Leone and the estimation of national transmission of EVD provides little information about local dynamics. To investigate district-level transmissibility of EVD, we applied a statistical modelling approach to estimate the basic reproduction number (*R*_0_) for each affected district and each country using weekly incident case numbers. We estimated growth rates during the early exponential phase of the outbreak using exponential regression of the case counts on the first eight weeks since onset. To take into account the heterogeneity between and within countries, we fitted a mixed effects model and calculated *R*_0_ based on the predicted individual growth rates and the reported serial interval distribution. At district level, *R*_0_ ranged from 0.36 (Dubréka) to 1.72 (Beyla) in Guinea, from 0.53 (Maryland) to 3.37 (Margibi) in Liberia and from 1.14 (Koinadugu) to 2.73 (Western Rural) in Sierra Leone. At national level, we estimated an *R*_0_ of 0.97 (95% CI 0.77–1.18) for Guinea, 1.26 (95% CI 0.98–1.55) for Liberia and 1.66 (95% CI 1.32–2.00) for Sierra Leone. Socio-demographic variables related to urbanisation such as high population density and high wealth index were found positively associated with *R*_0_ suggesting that the consequences of fast urban growth in West Africa may have contributed to the increased spread of EVD.

## Author Summary

Since 2013, a major epidemic of Ebola virus disease (EVD) spread throughout three countries in West Africa for about two years. Its scale was unprecedented since the Ebola virus was first identified. Almost all districts of Guinea, Liberia and Sierra Leone were affected, but some areas observed much larger outbreaks than others. In this study, we offer insights into the geographical variation of EVD transmissibility. We estimated the epidemic growth rate for each subnational unit and used the results to calculate the basic reproduction number *R*_0_, which indicates how many secondary infections arise on average from one primary case at the beginning of an outbreak. We found that the transmissibility of EVD differed within the three countries and we identified areas of high initial transmission. We also show that socio-demographic factors related to urbanisation and crowding might have been acting as drivers of EVD spread. These findings confirm that early containment of outbreaks in large communities is crucial for the prevention of epidemics as seen in West Africa during 2013–2015.

## Introduction

The Ebola virus disease (EVD) outbreak that started in December 2013 in Guinea developed into the largest EVD epidemic ever observed. There has been some discussion about the geographical heterogeneity of disease transmission in the three hardest hit countries in West Africa [1–8], but other studies have not considered this effect in their analysis. An epidemic of this scale has an intrinsic multi-level structure and national epidemic curves are always an overlay of local outbreaks [9–11]. Consequently, the estimation of national transmission offers little information about subnational dynamics. The availability of district-level data provides a unique opportunity to investigate local disease transmission during the 2013–2015 epidemic. We may also use these data to quantify population-level risk factors for EVD transmission. Demographic or behavioural factors such as crowding and high population density, low socioeconomic status (SES), unsafe burials or poor sanitation but also climate effects might have contributed to enhanced EVD transmission in West Africa, but these effects have been assessed only in a limited number of studies [1,7,11,12]. In the context of recurrent infectious diseases, increased human mobility and globalisation the quantification of subnational spread of EVD and the investigation of factors related to the spread might provide insights that could improve epidemic management in the future [5].

The most frequently used parameter for quantifying transmissibility is the basic reproduction number *R*_0_, which describes the average number of secondary infections generated by a primary case during the initial phase of an outbreak when the population is completely susceptible and no control measures have been employed [13]. A common approach to the estimation of *R*_0_ consists of fitting mathematical transmission models to observed outbreak data. In the West African EVD epidemic, several mathematical modelling studies have described the variation in *R*_0_ between [6,10,14–19] and within [20–25] the three countries. Most studies found estimates of *R*_0_ ranging between 1.5 and 2.5, agreeing with results from models of earlier outbreaks ranging from 1.4–4.7 (summarised in [26]). A drawback of mechanistic models is the requirement of a large number of parameters, which is problematic with sparse data [27]. As an alternative to mechanistic models, *R*_0_ can be inferred from the generation time and the intrinsic growth rate *r* (sometimes also referred to as Ʌ) during the early, exponential epidemic phase [13]. The generation time is the average interval between infection of a primary and a secondary case. The growth rate *r* is defined as the per capita change in case numbers per time unit. It can be calculated directly from the observed empirical data by estimating the slope of the natural log-transformed cumulative case counts over time with a linear regression model [28,29] or by fitting an exponential or sigmoid curve to incident case counts [27,30]. The use of incident case data is generally preferred over cumulative case data because the individual observations are statistically independent [1]. Ignoring the dependence of measurement error in cumulative cases can lead to over-optimistic standard errors and thus underestimation of uncertainty in epidemic growth [31]. This statistical approach is particularly useful for the assessment of smaller outbreaks with sparse data [27], as observed in certain districts during the West African EVD epidemic. The analysis of local transmissibility should take into account the hierarchical nature of subnational data, because these cases are not completely independent but arise from a common population i.e. country. Such an epidemic structure can be approached with a mixed effects model, which explicitly allows for clustering.

In this study, we used district-level case data from the Ebola epidemic in West Africa and implemented a statistical modelling approach to calculate the epidemic growth at country and at district level. We then estimated the transmissibility of EVD expressed as *R*_0_ for Guinea, Liberia and Sierra Leone and their 53 affected districts. Finally, we explored the relationship between *R*_0_ and socio-demographic variables as potential drivers of EVD transmission at a population-level.

## Methods

### EVD case data

We used data on weekly incident cases of EVD in each subnational unit (here called 'district') of Guinea (préfécture), Liberia (county) and Sierra Leone (district), which are available at the Ebola data and statistics website of the World Health Organization (WHO) [32]. These weekly case counts were aggregated from the patient database and are considered to be more reliable for the early phase of the epidemic than the situation reports issued by the Ministries of Health of the affected countries [33]. We calculated the total incident cases for each week as the sum of confirmed and probable incident cases. To take account of recurrent outbreaks we defined epidemic waves within each district. A wave was considered terminated after a period of 42 days with no new cases, corresponding to twice the maximum incubation period [34]. New cases arising after this period were considered as a new wave. To ensure a minimum number of data points in each district for the fitting process, we restricted our analysis to the first wave with three or more non-zero data points. Districts with fewer than three non-zero data points per wave were excluded. We restricted the time variable to the first eight weeks since onset of a wave in each district, corresponding to approximately four serial intervals [34]. This time period was considered long enough to have a sufficient number of time points and reduce initial stochasticity, but short enough to capture only the initial exponential phase and exclude the effects of control measures or natural attenuation of the epidemic. The comparison of the dates of epidemic onset and the opening dates of Ebola Treatment Units (ETUs, see [35,36]) showed that only 9% of all districts had a functioning ETU in the first eight weeks since onset of the selected wave.

### Mixed effects model for the estimation of the epidemic growth rates

We assumed that the epidemic growth in individual districts varied both between countries and between districts within a country. We used a generalised linear mixed effects model (GLMM) comprising both fixed and random effects, which explicitly allows for clustering in the data [37]. Such a hierarchical model allows the mean values to vary between the different countries, but borrows information across the districts within a country. The weekly number of new infections *c*_*ijk*_ in district *i* in country *j* at time-point *t*_*ijk*_ was assumed to follow a Poisson distribution with a mean λ_ijk_ and was modelled with the logarithm as the link function:

$$c_{ijk}\sim \;Pois\left(\lambda_{ijk}\right)$$

$$\text{ln}\left(\lambda_{ijk}\right)=(\beta_{0j}+b_{0ij})+\left(\beta_{1j}+b_{1ij}\right)*t_{ijk}$$

$$\left(\begin{array}{c} b_{0ij}\\ b_{1ij} \end{array}\right)\;\sim \;N_{2}\left(\left(\begin{array}{c} 0\\ 0 \end{array}\right),\left(\begin{array}{cc} \tau_{0}^{2} & \rho \tau_{0}\tau_{1}\\ \rho \tau_{0}\tau_{1} & \tau_{0}^{2} \end{array}\right)\right)$$ (1)

This means we assume country level intercepts *β*_0*j*_ and slopes *β*_1*j*_, which are modelled as fixed effects. The district specific intercepts *β*_0*j*_ *+ b*_0ij_ and slopes *β*_1*j*_ *+ b*_1ij_ are assumed to be normally distributed around these country level average values. For simplicity, we assume the heterogeneity between the district-level intercepts (*τ*_0_^2^) and slopes (*τ*_1_^2^) as well as their correlation (ρ) to be the same for all countries. The model was fitted using maximum likelihood estimation. We then used the posterior means of the random effects (empirical Bayes means) to calculate the growth rates *r* for each district *i* in country *j*, given by

$$r_{ij}\;=\;\beta_{1j}\;+\;b_{1ij}$$ (2)

### Basic reproduction number *R*_0_

The expression relating *r* to *R*_0_ is the inverse of the moment generating function of the generation time distribution, which uniquely identifies *R*_0_ for a given *r* and generation time distribution [13]. If the generation time is known and if it follows a gamma distribution, *R*_0_ can be calculated as

$$R_{0}={\left(1+\frac{r}{\beta }\right)}^{\alpha }$$ (3)

where *r* is the intrinsic growth rate, *α* is the shape parameter and *β* is the rate parameter of the gamma distribution, respectively [13]. For known *α* and *β*, we can derive the uncertainty in *R*_*0*_ from the uncertainty in the growth rate estimates *r* with the delta method [37]. The variance (Var) of *R*_*0*_ is approximated as

$$\text{Var}\left(R_{0}\left(r\right)\right)=\text{Var}\left(r\right)*{\left(\frac{\partial R_{0}\left(r\right)}{\partial r}\right)}^{2}$$ (4)

and the standard error (SE) is the square root of the variance. The lower and upper bounds of an approximate 95% confidence interval (CI) are then calculated as the estimate±1.96*SE derived with the delta method. The shape (2.59) and rate (0.17 per day) parameters of the serial interval were obtained by fitting a gamma distribution to the serial interval distribution reported by the WHO Ebola response team [34]. For simplicity, we did not consider the uncertainty in the estimation of *α* and *β* (which could be done by applying multivariate versions of the delta method).

The estimated epidemic growth rate might be influenced by the length of the time window under investigation. To examine the impact of assumptions about the exponential phase on our estimates, we performed a sensitivity analysis for the mixed effects model with time windows one to three weeks shorter (five to seven weeks) and longer (nine to eleven weeks) than the proposed eight weeks.

### Associations between *R*_*0*_ and socio-demographic factors

We used an ecological study design to quantify the potential impact of risk factors for EVD transmission at district level. We focused on variables that are strongly suspected to be drivers of infectious disease spread: population density, household density, low SES and poor sanitation. Population density was calculated using population sizes and surface areas derived from national censuses [38–40], the other variables were derived as district-level summary measures from Demographic and Health Survey (DHS) datasets [41–43]. Household density was calculated as the number of persons per sleeping room. SES was approximated using the DHS wealth index, which is a composite score of household assets on a continuous scale transformed to a standard normal distribution for each country. An increasing score indicates a higher SES. For simplicity, we did not calculate a comparative wealth index score [44], which generally limits the comparability of the SES across countries, but we consider the use of the original score adequate for the purpose of this analysis. We used the average time to walk to the nearest water source as a proxy for the level of sanitation. The calculation of the summary estimates is described in detail in the supplement (S1 Text).

### Statistical analysis

All analyses were carried out using Stata (StataCorp. 2013. Stata Statistical Software: Release 13. College Station, TX: StataCorp LP). The mixed effects model was implemented using the meglm routine with the unstructured covariance option. The association between socio-demographic factors and transmissibility as measured by *R*_*0*_ was quantified in a univariable linear regression model. All summary data are expressed as median and range or mean and standard deviation (SD). Differences between countries were tested using one-way analysis of variance (ANOVA).

## Results

### EVD case data

**Fig 1:**
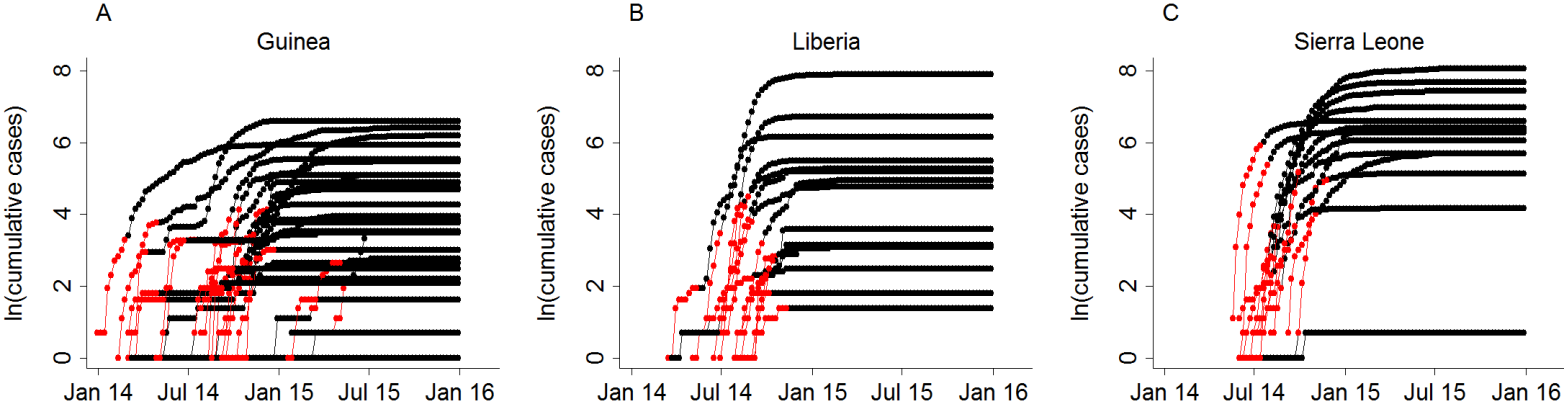
Natural log-transformed cumulative EVD case numbers by district and country. **A**. Guinea. **B**. Liberia. **C**. Sierra Leone. The red dots indicate the data points included in the mixed effects model.

We used recent WHO datasets for Guinea, Liberia and Sierra of May 11, 2016, which contained 4391 data points for 56 affected of a total of 63 districts for the years 2013–2015. Three districts were excluded because they had fewer than three non-zero data points in one wave (Dinguiraye, Guinea; Togué, Guinea and Bonthe, Sierra Leone). The restriction of the dataset to the first 8 weeks since onset resulted in 477 data points for 53 districts (nine data points per district). For most districts, the first epidemic wave was large enough to be included in the analysis, except for seven districts where we used the second wave (Boké, Fria, Kindia and Siguiri in Guinea; Margibi and Nimba in Liberia and Koinadugu in Sierra Leone) and one district where we used the fourth wave (Kouroussa in Guinea). The different epidemic waves in each district are displayed in the supplement (S1 Fig). On visual inspection, the restriction of the time variable captured the initial growth phase appropriately (Fig 1).

### Basic reproduction number *R*_0_

**Fig 2:**
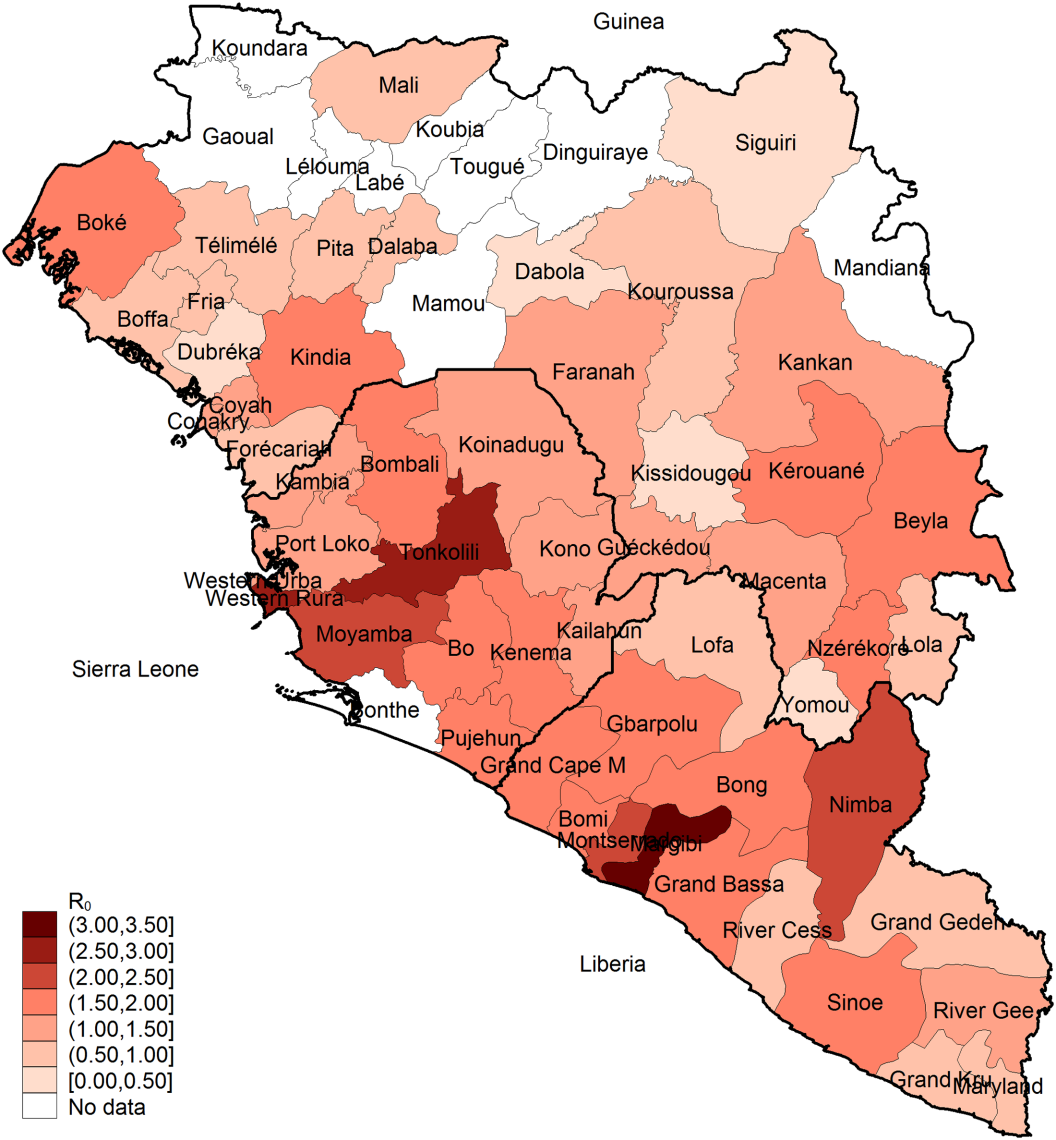
Geographical distribution of district-level *R*_0_. The shapefiles were retrieved from the Database of global administrative areas GADM ([45]).

**Fig 3:**
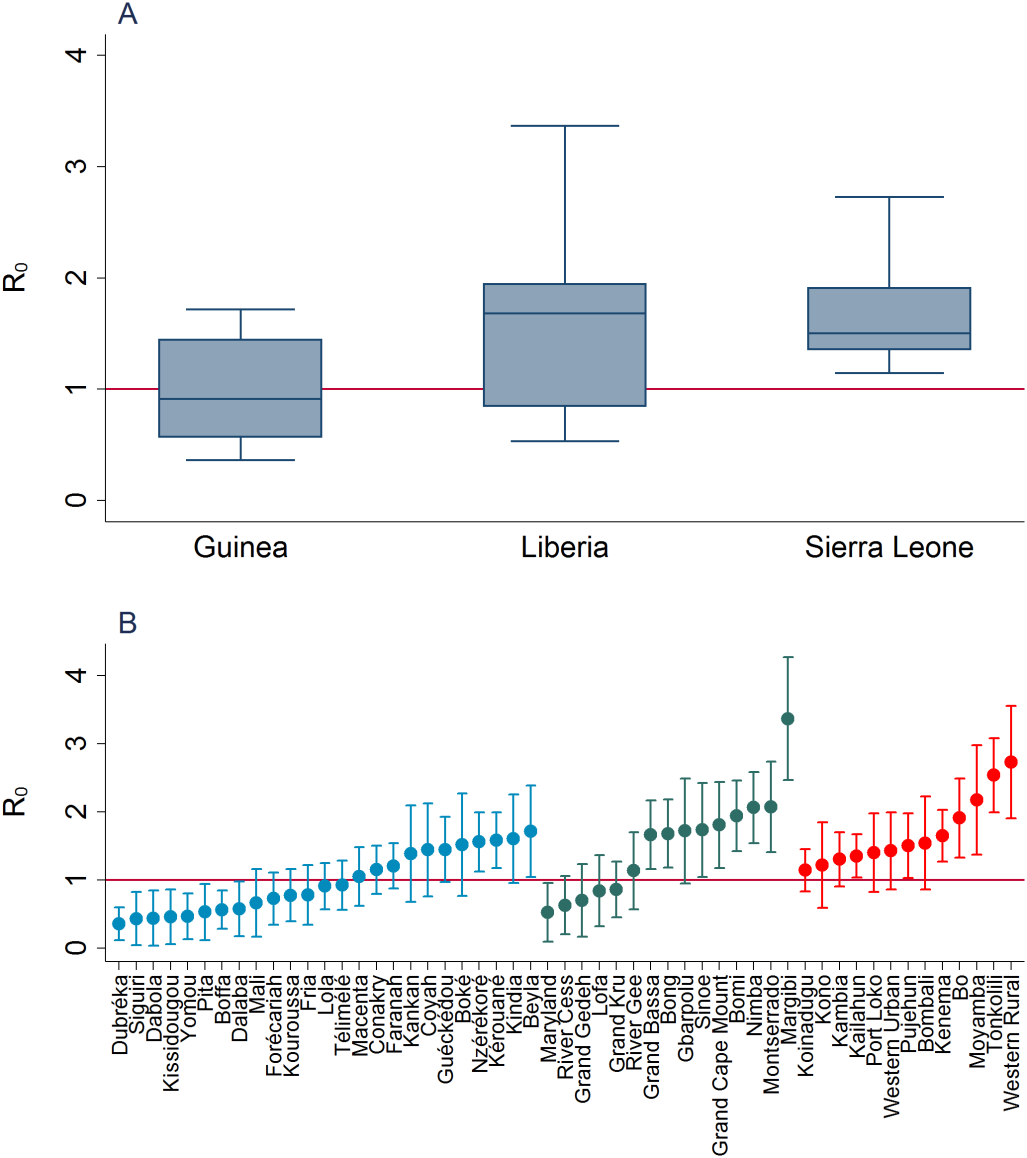
District-level estimates of *R*_0_. The red horizontal line indicates the epidemic threshold of *R*_0_ = 1. **A**. The box shows the interquartile range, the horizontal line is the country median. The ends of the whiskers are the lower and upper range values. **B**. Individual estimates and 95% confidence intervals of *R*_0_ for districts in Guinea (blue), Liberia (green) and Sierra Leone (red).

Epidemic growth rates and thus *R*_0_ differed at both district and country level. The spatial distribution of district-level estimates shows that districts with high transmissibility appear to cluster regionally irrespective of national borders (Fig 2). At district level, *R*_0_ ranged from 0.36 (Dubréka) to 1.72 (Beyla) in Guinea, from 0.53 (Maryland) to 3.37 (Margibi) in Liberia and from 1.14 (Koinadugu) to 2.73 (Western Rural) in Sierra Leone (Table 1). Transmissibility was below the epidemic threshold of *R*_0_ = 1 in 56% of all districts in Guinea, in 33% of all district in Liberia and in none of the districts in Sierra Leone (Fig 3). District-level *R*_0_ values differed not more between than within the three countries (one-way ANOVA, F(2, 50) = 8.38, p = 0.083). The values of *R*_0_ and 95% CIs for each district are provided in the supplement (S1 Table). We also estimated a national *R*_0_ of 0.97 (95% CI 0.77–1.18) for Guinea, 1.26 (95% CI 0.98–1.55) for Liberia and 1.66 (95% CI 1.32–2.00) for Sierra Leone. The overall mean of the district-level *R*_0_ values was 1.30 (SD 0.64) and the distribution was right-skewed (S2 Fig).

**Table 1:** District-level and national estimates of *R*_0_.

	Guinea (N = 25)		Liberia (N = 15)		Sierra Leone (N = 13)	
**Districts**†	**Median**	**(range)**	**Median**	**(range)**	**Median**	**(range)**
***R***_**0**_	0.91	(0.36–1.72)	1.68	(0.53–3.37)	1.50	(1.14–2.73)
National*	**Estimate**	**(95% CI)**	**Estimate**	**(95% CI)**	**Estimate**	**(95% CI)**
***R***_**0**_	0.97	(0.77–1.18)	1.26	(0.98–1.55)	1.66	(1.32–2.00)

^†^ The district-level growth rate estimates correspond to the sum of the country-specific fixed effect and the district-specific random effect

* The national growth rate estimates correspond to the fixed effects of the GLMM

The inclusion of shorter or longer time windows for the mixed effects model had little influence on the magnitude of the national estimates of *r* and *R*_0_, but as expected the uncertainty in the estimate increased with decreasing length of the time window (S2 Table and S3 Fig). At district level, the median did not vary substantially in Guinea, but some districts showed more extreme estimates for time windows of five weeks or more than nine weeks. In Liberia and Sierra Leone the median decreased towards one for time windows of five weeks or more than ten weeks.

### Associations between *R*_0_ and socio-demographic factors

**Fig 4:**
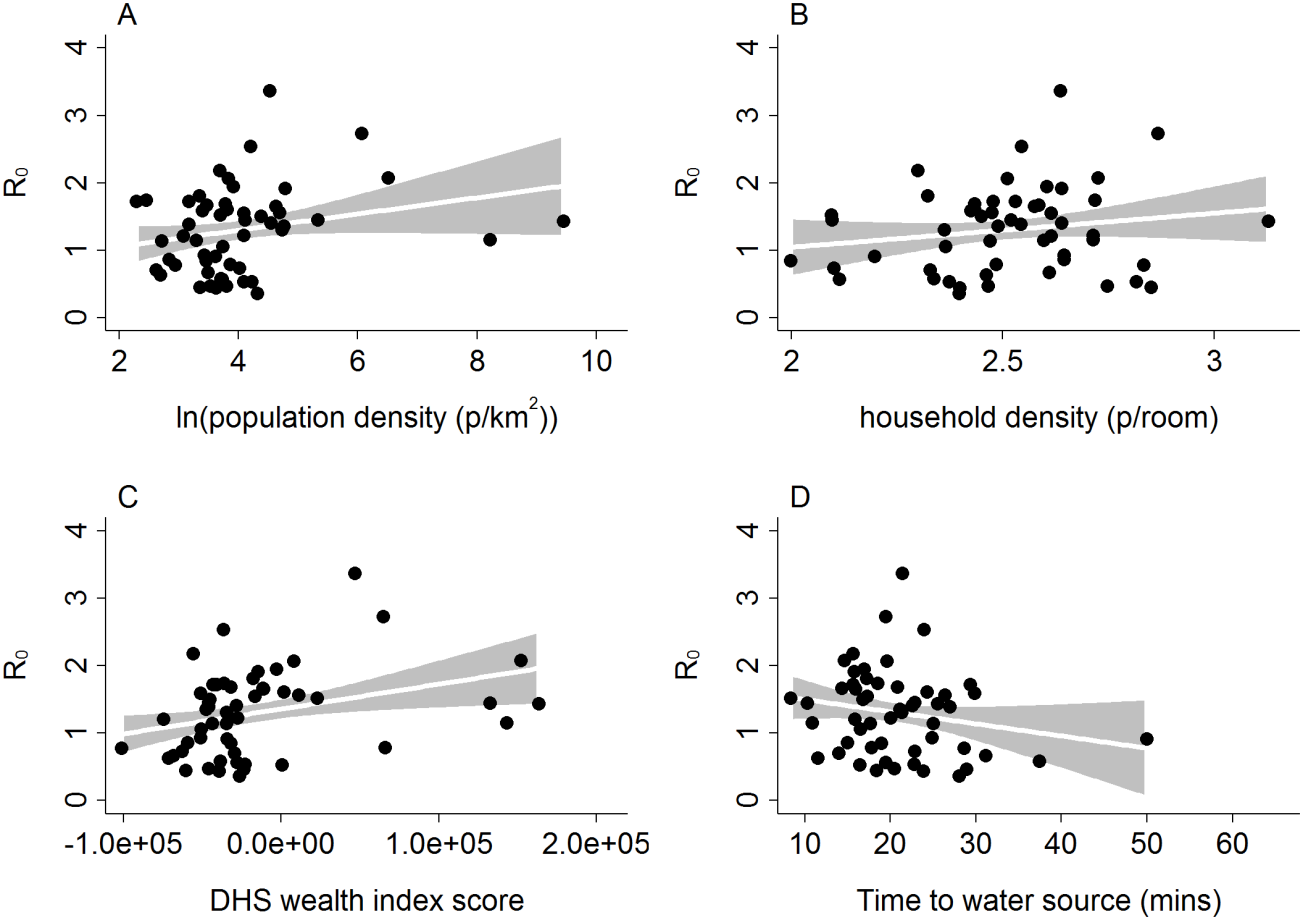
Scatterplots of *R*_0_ and socio-demographic exposure variables. **A**. Association between *R*_0_ and log-transformed population density (p/km^2^). **B**. Association between *R*_0_ and household density (p/room). **C**. Association between *R*_0_ and DHS wealth index score. **D**. Association between *R*_0_ and time to water source (mins). The white line denotes the linear fit, the grey shaded area denotes the 95% CI. DHS = Demographic and Health Survey, p = persons.

We found no association between *R*_0_ and household density (β = 0.51, 95% CI -0.30–1.31, p = 0.215) or time taken to walk to the nearest water source (β = -0.18, 95% CI -0.43–0.07, p = 0.151) (Table 2 and Fig 4). There was weak statistical evidence for a positive association between log-transformed population density and *R*_0_ (β = 0.12, 95% CI -0.02–0.26, p = 0.086). The DHS wealth index score showed the strongest statistical evidence for a positive association with *R*_0_ (β = 0.37, 95% CI 0.07–0.67, p = 0.017). This result appears counterintuitive, but an increasing wealth score was also positively associated with population density suggesting that both variables act as a proxy for urbanisation and its effects on human contact patterns. These findings suggest that variables associated with large-scale crowding may act as population-level risk factors for EVD transmission.

**Table 2:** Association between socio-demographic exposure variables and *R*_0_ in a linear univariable model.

Variable	Unit	β	(95% CI)	p-value
Population density	per log_e_	0.12	(-0.02–0.26)	0.086
Household density	per person	0.51	(-0.30–1.31)	0.215
DHS wealth score	per 10^5^	0.37	(0.07–0.67)	0.017
Time to water source	per 10 minutes	-0.18	(-0.43–0.07)	0.151

DHS Demographic and Health Survey, p-value from Wald test

## Discussion

Our study provides further evidence that the EVD epidemic in West Africa was a spatially heterogeneous process at district level, and that socio-demographic factors might have contributed to the spread of EVD. Average district transmissibility was lower than the epidemic threshold of *R*_0_ = 1 in Guinea but higher in Liberia and Sierra Leone. Geographically adjacent areas appeared to have a similar transmissibility regardless of country borders. The spatial distribution of transmission estimates suggested a cluster region in the coastal districts in the southwest of Sierra Leone and Liberia and in the east of Guinea and Liberia, and less intense transmission in the north of Guinea. Population density and a high DHS wealth index score at district-level were positively associated with *R*_0_ suggesting that factors related to urbanisation and large-scale crowding might have contributed to the rapid spread of EVD in certain areas.

The strength of our statistical approach is that it allowed for a more realistic scenario of growth rates than models that treat each outbreak individually. The use of a generalised linear mixed model provides a simple but elegant solution for a geographically complex and hierarchical epidemic structure and yielded plausible values of transmissibility. As shown in our sensitivity analysis, the length of the time window for the exponential phase did not affect the estimates of *R*_0_ substantially. The selection of the time interval to be included should be based on considerations about the generation time, the number of data points available and knowledge about the time point of control interventions. Our approach has three main limitations, two related to data quality and one to the choice of methods. First, the reliability of the data collected at the beginning of the epidemic is uncertain [46]. Reporting delays and underreporting could have led to the underestimation of the incidence. Delays in updating the patient database do not affect our analysis because we included only the initial data points for each district. Underreporting of cases was estimated to be 17 to 70% [47]. If underreporting remains constant throughout the observed time period, *R*_0_ is unaffected, because the exponential growth rate does not change. The assumption of proportional underreporting seems reasonable for the initial phase of two months even if it does not hold throughout the epidemic. Due to the lack of data on dynamic underreporting, we did not consider this aspect in our analysis. Second, our assumption about the absence of control measures might not be true. Local outbreaks occurred at different time points of the epidemic. Immediate implementation of control measures or increased public awareness in later outbreaks could have biased estimates of the initial epidemic growth downwards for districts affected towards the end of 2014. We think that the effect of these interventions was negligible during the first two months of an outbreak because, even by early October 2014, only a few districts had managed to implement fully functioning control measures such as safe burials and contact tracing [48] and most ETUs opened more than eight weeks after onset of the epidemic in a district. Third, our approach does not take into account the spatial dependence of cases and in theory the geographical location of cases is exchangeable within a country in our model. Spatial autocorrelation models require a spatial weights matrix, which is derived using geographical information, and are conceptually more complex than normal regression models. We aimed to establish a simple model, which can be used when limited geographical information is available but which still includes the hierarchical aspect of spatially heterogeneous epidemics. This analysis also has a limited spatial resolution due to the availability of data and cannot capture heterogeneities at a finer scale. The assumption of homogeneous population mixing within a district is clearly unrealistic. However, the trade-off between higher spatial resolution and lower case numbers is not straightforward. Chiefdom-level data may provide a better picture of local dynamics but are not publicly available.

Our district-level estimates of *R*_0_ are generally consistent with results of studies from Kenema (Sierra Leone), Montserrado (Liberia) and Conakry (Guinea) (S3 Table). Four studies using different phylodynamic models based on patient samples taken in a hospital in Kenema have given estimates of *R*_0_ between 1.26 and 2.40 [23–25,47]. Our estimate of 1.65 (95% CI 1.27–2.03) is within this range. For Montserrado, we estimated an *R*_0_ of 2.07 (95% CI 1.41–2.74), which lies between the published estimates of 1.73 and 2.49 [21,22]. For Conakry, our model yielded a lower value (1.15, 95% CI 0.80–1.51) than that calculated from reconstructed transmission chains (1.7) for the time period of March 2014 [20] but was close to the overall value of 0.95 estimated from a re-analysis of the same data [49]. The finding that many districts experienced an outbreak that was characterised by an *R*_0_<1 supports the hypothesis of heterogeneous contact networks [4,11]. Clustered transmission and superspreading has been confirmed by several studies [20,25,47] and can lead to outbreaks even if *R*_0_ is below unity [49]. Assuming that our approach is an adequate model of this hierarchical epidemic, we may conclude that subnational transmissibility might not have been as high as previously thought. At national level, our approach was not comparable to results of other methods. Several mechanistic [6,10,14,16–19,50–52] and phenomenological [15,34,53] models have provided estimates of *R*_0_ ranging from 1.51 (95% CI 1.50–1.52) to 2.46 (95% CI 1.44–2.01) for Guinea, from 1.54 to 2.5 (95% CI 2.4–2.7) for Liberia and from 1.26 to 8.33 for Sierra Leone. Our estimates of 0.97 (95% CI 0.77–1.18) for Guinea, of 1.26 (95% CI 0.98–1.55) for Liberia and of 1.66 (95% CI 1.43–2.00) for Sierra Leone are consistently lower than all published values. These differences might reflect the fact that our national estimates of *R*_0_ result from an averaged growth rate of multiple local outbreaks occurring at different time points, whilst other studies used the national epidemic curves to fit their models.

The ecological analysis showed associations between *R*_*0*_ and population-level factors linked to urbanisation and crowding. Analysis at the population-level is appropriate when the mechanism of action involves interactions between individuals, for example the potential for spread of an infection in densely populated areas [54]. Nevertheless, the risk of an individual of becoming infected cannot be predicted from the population density of their household or community. Whether the positive association between crowding and EVD transmissibility is also observed at a lower level of administrative unit cannot be inferred from this analysis. Fallah et al used individual data on EVD cases and their contacts and determined SES-stratified measures of transmission [12]. They found that cases from middle and low SES communities caused significantly more secondary cases than infected individuals from high SES communities. These findings appear contradictory to our study, but can be explained by the different levels of data and different definitions of the SES. While our SES variable considers housing properties and household assets, Fallah et al also included high population density in the definition of low SES. Considering these differences, both studies are consistent with the hypothesis that large-scale crowding contributed to EVD transmission. Other factors that could influence the transmissibility could not be considered in this analysis due to lack of data. Behavioural factors such as community resistance or superspreading events like unsafe burials [55] might have had a strong impact on EVD spread. Some of our estimates may be large by chance due to the stochastic nature of the outbreaks. Biological factors such as differences in host or viral genetics are probably less important in this population and this time frame. Our findings are compatible with theory about drivers of infectious diseases, but the contribution of different factors cannot be answered completely with this study design and these data.

This study has shown that the mixed effects model is a suitable strategy to quantify local epidemic growth during a large-scale multifocal epidemic and confirms the notion of geographical heterogeneity in the transmission of EVD in West Africa. Social and demographic variables measured at the population level and related to urbanisation, such as high population density and high SES, were positively associated with *R*_0_ suggesting that the consequences of fast urban growth in West Africa may have contributed to the increased spread of EVD.

## Supporting Information

S1 Text Calculation of summary estimates from DHS datasets. (PDF)

S1 Fig Epidemic curves with distinct epidemic waves for each district. The colors denote the order of the waves: red = first wave, blue = second wave, green = third wave, magenta = fourth wave, black = interwave periods with no cases
(TIF)

S2 Fig Overall distribution of district-level *R*_0_. The histogram was calculated with a bin width of 0.1.
(TIF)

S3 Fig Influence of different time windows included in the mixed effects model on magnitude of *R*_0_. **A-C**. National estimates (line) and 95% confidence intervals (shaded area). **D-F**. District-level medians (line) and range (shaded area).
(TIF)

S1 Table Individual estimates and confidence intervals of *R*_0_ for each district. (XLS)

S2 Table Sensitivity analysis with varying time windows for national and district-level *R*_0_. (PDF)

S3 Table Comparison of other published estimates (95% confidence or credible intervals) of *R*_0_ (PDF)
