# Supplementary material for: Heterogeneity in District-Level Transmission of Ebola Virus Disease during the 2013-2015 Epidemic in West Africa
Source: PLoS Negl Trop Dis. 2016 Jul 19;10(7):e0004867. doi: 10.1371/journal.pntd.0004867 (PMC4951043; doi:10.1371/journal.pntd.0004867)
Supplement: S1 Text — (PDF) [file pntd.0004867.s001.pdf]

Heterogeneity in District-level Transmission of Ebola Virus Disease during the 2013-2015 Epidemic in West Africa

Fabienne Krauer, Sandro Gsteiger, Nicola Low, Christian H. Hansen and Christian L. Althaus

S1 Text: Calculation of summary estimates from DHS datasets

DHS are cross-sectional, nationally representative household surveys, which collect data on demographic, socioeconomic and health-related topics (http://www.dhsprogram.com/). We used the variables hv013 (number of *de facto* members in household), hv216 (number of rooms used for sleeping), hv204 (time to get to water source) and hv271 (wealth index score) of the household datasets. The wealth index is a composite score of a household's cumulative living standards [1]. It is generated based on several household variables with a principal component analysis (PCA) and places individual households on a continuous scale of relative wealth. The DHS uses a stratified sampling design, where the strata are the subnational regions of residence. In Liberia and Sierra Leone, the strata correspond to the districts and the variable could be used without further recoding. In Guinea, the strata correspond to the regions, which are at a lower spatial resolution than the préféctures. Therefore, all households were linked to the DHS georeferenced dataset, which contains the coordinates for each household, and were then assigned to the corresponding districts using QGIS (QGIS Geographic Information System, Version 2.6.1). Finally, we summarised all exposure variables as means at district-level using the appropriate weights and stratification. The application of weights adjusts for differences in sampling probability and response rates.

1. Rutstein SO, Johnson K. The DHS Wealth Index. DHS Comparative Reports No. 6. Calverton, Maryland, USA; 2014.
