# Supplementary figures and images for: Heterogeneity in District-Level Transmission of Ebola Virus Disease during the 2013-2015 Epidemic in West Africa
Source: PLoS Negl Trop Dis. 2016 Jul 19;10(7):e0004867. doi: 10.1371/journal.pntd.0004867 (PMC4951043; doi:10.1371/journal.pntd.0004867)

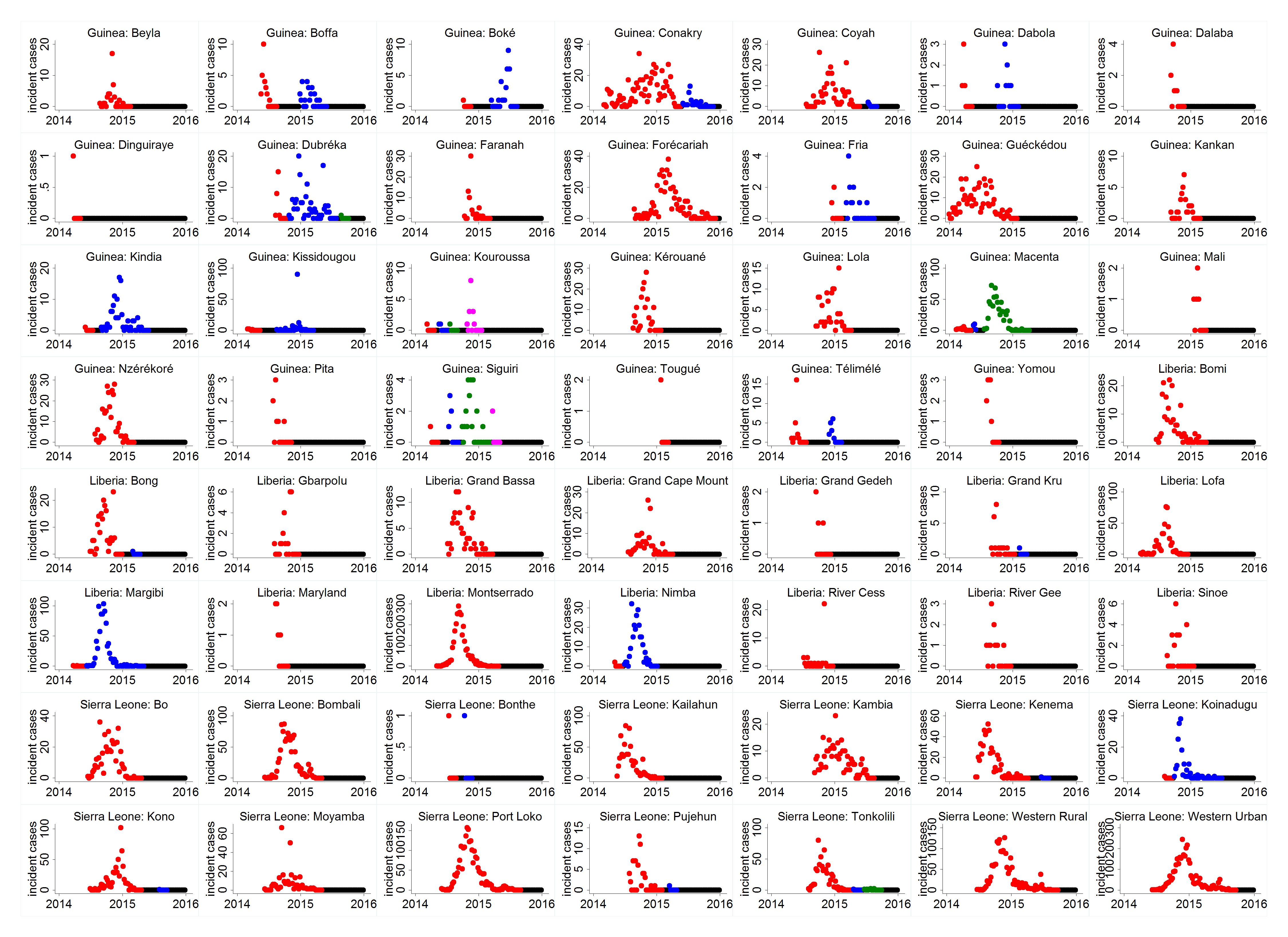

Supplement: S1 Fig — The colors denote the order of the waves: red = first wave, blue = second wave, green = third wave, magenta = fourth wave, black = interwave periods with no cases (TIF) [file pntd.0004867.s002.tif]

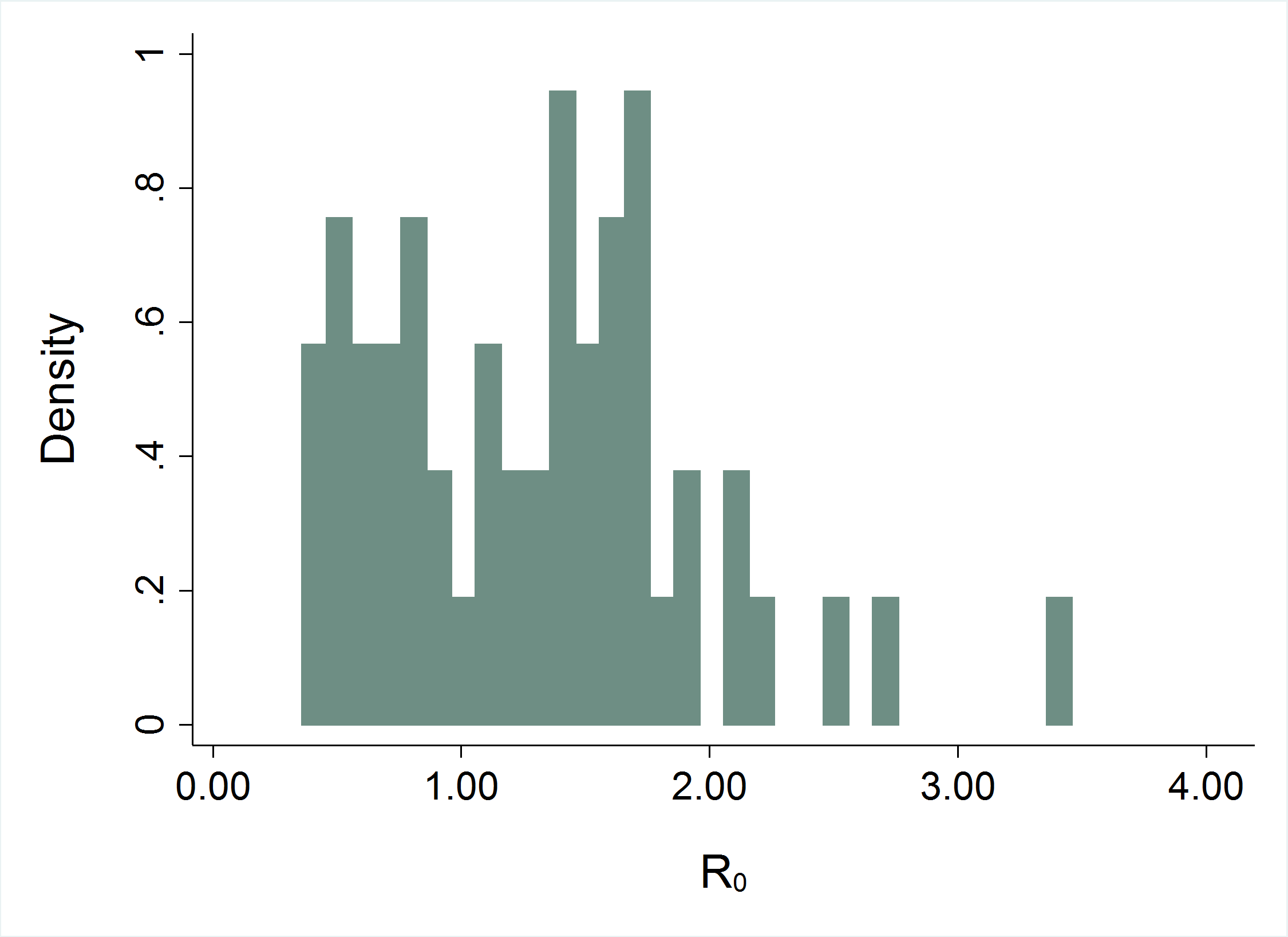

Supplement: S2 Fig — The histogram was calculated with a bin width of 0.1. (TIF) [file pntd.0004867.s003.tif]

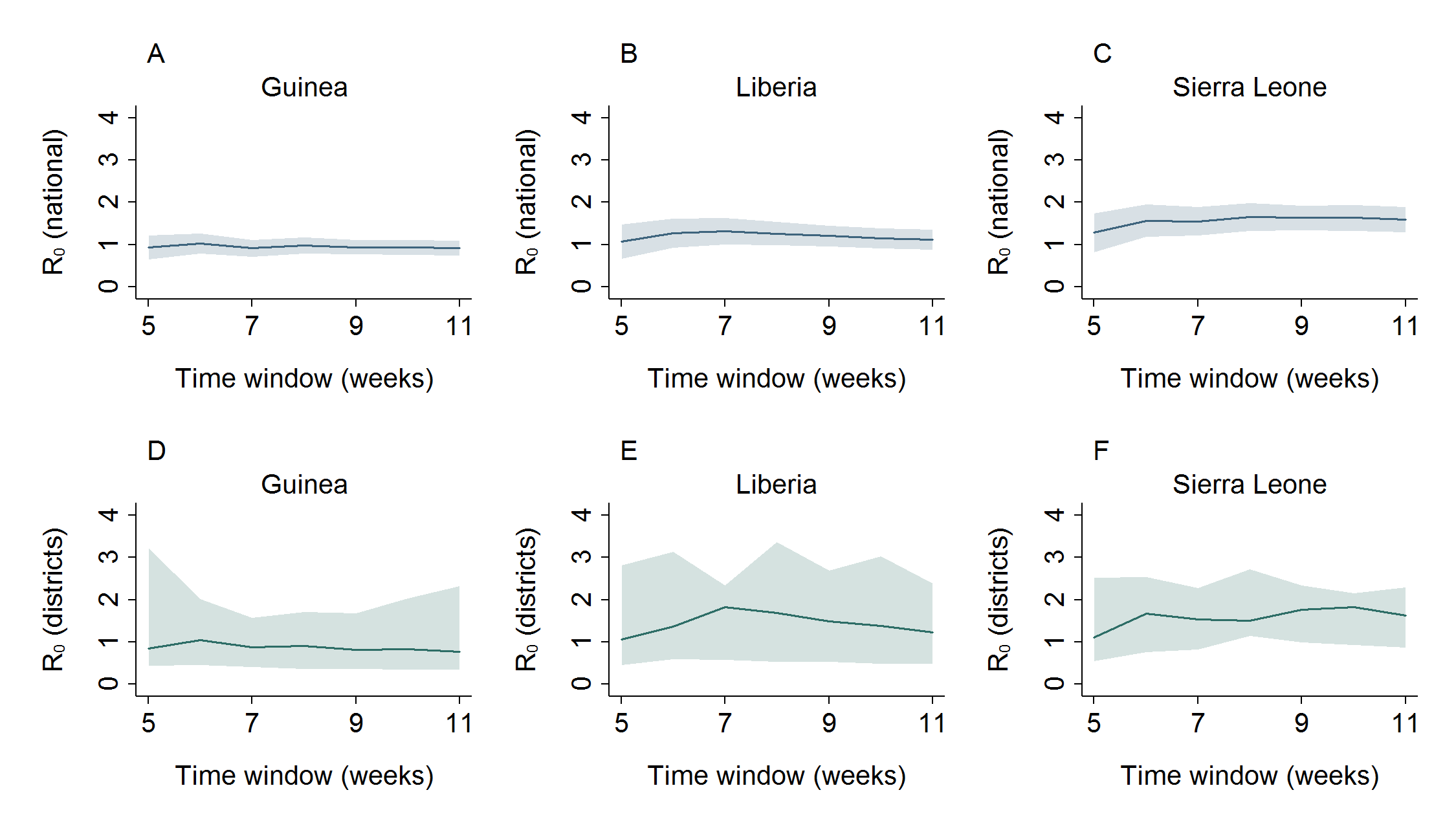

Supplement: S3 Fig — A-C. National estimates (line) and 95% confidence intervals (shaded area). D-F. District-level medians (line) and range (shaded area). (TIF) [file pntd.0004867.s004.tif]
