# Supplementary material for: Heterogeneity in District-Level Transmission of Ebola Virus Disease during the 2013-2015 Epidemic in West Africa
Source: PLoS Negl Trop Dis. 2016 Jul 19;10(7):e0004867. doi: 10.1371/journal.pntd.0004867 (PMC4951043; doi:10.1371/journal.pntd.0004867)
Supplement: S2 Table — (PDF) [file pntd.0004867.s006.pdf]

Heterogeneity in District-level Transmission of Ebola Virus Disease during the 2013-2015 Epidemic in West Africa

Fabienne Krauer, Sandro Gsteiger, Nicola Low, Christian H. Hansen and Christian L. Althaus

## S2 Table. Sensitivity analysis with varying time windows for national and district-level $R_0$

| **Time window** | **Guinea (N=25)** | | **Liberia (N=15)** | | **Sierra Leone (N=13)** | |
|---|---|---|---|---|---|---|
| **Districts** | **Median** | **(range)** | **Median** | **(range)** | **Median** | **(range)** |
| 5 weeks | 0.84 | (0.43-3.24) | 1.06 | (0.45-2.82) | 1.10 | (0.55-2.53) |
| 6 weeks | 1.04 | (0.45-2.03) | 1.37 | (0.58-3.14) | 1.67 | (0.76-2.55) |
| 7 weeks | 0.88 | (0.40-1.58) | 1.82 | (0.58-2.35) | 1.54 | (0.82-2.28) |
| 8 weeks* | 0.91 | (0.36-1.72) | 1.68 | (0.53-3.37) | 1.50 | (1.14-2.73) |
| 9 weeks | 0.82 | (0.35-1.70) | 1.49 | (0.52-2.71) | 1.76 | (0.99-2.34) |
| 10 weeks | 0.83 | (0.34-2.05) | 1.38 | (0.47-3.03) | 1.82 | (0.92-2.17) |
| 11 weeks | 0.77 | (0.34-2.34) | 1.23 | (0.47-2.40) | 1.62 | (0.86-2.31) |
| **National** | **Estimate** | **(95% CI)** | **Estimate** | **(95% CI)** | **Estimate** | **(95% CI)** |
| 5 weeks | 0.93 | (0.64-1.22) | 1.08 | (0.66-1.49) | 1.28 | (0.81-1.75) |
| 6 weeks | 1.03 | (0.79-1.27) | 1.27 | (0.92-1.63) | 1.57 | (1.18-1.96) |
| 7 weeks | 0.92 | (0.71-1.12) | 1.32 | (1.00-1.64) | 1.55 | (1.20-1.90) |
| 8 weeks* | 0.97 | (0.77-1.18) | 1.26 | (0.98-1.55) | 1.66 | (1.32-2.00) |
| 9 weeks | 0.94 | (0.76-1.12) | 1.20 | (0.95-1.46) | 1.63 | (1.33-1.94) |
| 10 weeks | 0.93 | (0.75-1.12) | 1.15 | (0.90-1.40) | 1.63 | (1.32-1.95) |
| 11 weeks | 0.92 | (0.74-1.10) | 1.12 | (0.88-1.36) | 1.59 | (1.29-1.90) |

*8 weeks corresponds to the time window of the primary analysis
