# Supplementary material for: Heterogeneity in District-Level Transmission of Ebola Virus Disease during the 2013-2015 Epidemic in West Africa
Source: PLoS Negl Trop Dis. 2016 Jul 19;10(7):e0004867. doi: 10.1371/journal.pntd.0004867 (PMC4951043; doi:10.1371/journal.pntd.0004867)
Supplement: S3 Table — (PDF) [file pntd.0004867.s007.pdf]

Heterogeneity in District-level Transmission of Ebola Virus Disease during the 2013-2015 Epidemic in West Africa

Fabienne Krauer, Sandro Gsteiger, Nicola Low, Christian H. Hansen and Christian L. Althaus

S3 Table. Comparison of other published estimates (95% confidence or credible intervals) of R_0

Type of model	Guinea	Liberia	Sierra Leone	Reference
**National**				
SEIR	1.51 (1.50-1.52)	1.59 (1.57-1.60)	2.53 (2.41-2.67)	[1]
IDEA	2.46	1.72	8.33	[2]
growth rate		1.96 (1.92-2.01)	3.07 (2.85-3.32)	[3]
growth rate	1.71 (1.44-2.01)	1.83 (1.72-1.94)	2.02 (1.79-2.26)	[4]
extended SEIR		2.22	1.78	[5]
extended SEIR		1.63 (1.59-1.66)		[6]
extended SEIR		1.54	1.26	[7]
extended SEIR		1.84 (1.60-2.13)		[8]
extended SEIR			2.13	[9]
extended SEIR		1.76	1.49	[10]
stochastic ABM*		2.5 (2.4-2.7)	2.5 (2.4-2.7)	[11]
extended SEIR		2.11 (1.88-2.71)		[12]
extended SEIR		2.01		[13]
**Subnational**				
**Conakry, Guinea**				
transmission tree	1.7 (1.2-2.3)			[14]
**Montserrado, Liberia**				
stochastic		1.73 (1.66-1.83)		[15]
extended SEIR		2.49 (2.38-2.60)		[16]
**Kenema, Sierra Leone**				
phylodynamic BDEI			2.18 (1.24-3.55)	[17]
phylodynamic BD			1.26 (1.04-1.54)	[18]
phylodynamic SEIIR			2.40 (1.54-3.87)	[19]
phylodynamic SEIR			1.40 (1.1-1.8)	[20]

SEIR susceptible-exposed-infected-recovered model, IDEA incidence decay and exponential adjustment model, ABM agent-based model, BDEI birth-death-exposed-infected model, BD birth-death model
*corresponds to R_e for the early period of May 27-August 4, 2014

References

1. Althaus CL. Estimating the Reproduction Number of Ebola Virus (EBOV) During the 2014 Outbreak in West Africa. PLoS Curr. 2014;6(1):1–9.
2. Fisman D, Khoo E, Tuite A. Early epidemic dynamics of the west african 2014 ebola outbreak: estimates derived with a simple two-parameter model. PLoS Curr. 2014;6(1).
3. Chowell G, Nishiura H. Transmission dynamics and control of Ebola virus disease (EVD): a review. BMC Med. 2014 Jan 21;12(1):196.
4. WHO Ebola Response Team. Ebola virus disease in West Africa--the first 9 months of the epidemic and forward projections. N Engl J Med. United States; 2014 Sep 22;371(16):1481–95.
5. Rivers C, Lofgren E, Marathe M, Eubank S, Lewis B. Modeling the Impact of Interventions on an Epidemic of Ebola in Sierra Leone and Liberia. PLoS Curr. 2014;Oct 16(1).
6. Pandey A, Atkins KE, Medlock J, Wenzel N, Townsend JP, Childs JE, et al. Strategies for containing Ebola in West Africa. Science. 2014 Nov 21;346(6212):991–5.
7. Webb G, Browne C, Huo X, Seydi O, Seydi M, Magal P. A Model of the 2014 Ebola Epidemic in West Africa with Contact Tracing. PLoS Curr. 2015;1–10.
8. Merler S, Ajelli M, Fumanelli L, Gomes MFC, Pastore y Piontti A, Rossi L, et al. Spatiotemporal spread of the 2014 outbreak of Ebola virus disease in Liberia and the effectiveness of non-pharmaceutical interventions: a computational modelling analysis. Lancet Infect Dis. 2015;15(2):204–11.
9. White RA, MacDonald E, de Blasio BF, Nygård K, Vold L, Røttingen J-A. Projected Treatment Capacity Needs in Sierra Leone. PLoS Curr. 2014;1–16.
10. Khan A, Naveed M, Dur-E-Ahmad M, Imran M. Estimating the basic reproductive ratio for the Ebola outbreak in Liberia and Sierra Leone. Infect Dis poverty. London; 2015;4(13):13.
11. Siettos C, Anastassopoulou C, Russo L, Grigoras C, Mylonakis E. Modeling the 2014 Ebola Virus Epidemic – Agent-Based Simulations, Temporal Analysis and Future Predictions for Liberia and Sierra Leone. PLoS Curr. 2015;(December 2013):1–16.
12. Valdez LD, Aragão Rêgo HH, Stanley HE, Braunstein LA. Predicting the extinction of Ebola spreading in Liberia due to mitigation strategies. Sci Rep. 2015;5:12172.
13. Xia Z-Q, Wang S-F, Li S-L, Huang L-Y, Zhang W-Y, Sun G-Q, et al. Modeling the transmission dynamics of Ebola virus disease in Liberia. Sci Rep. 2015;5:13857.
14. Faye O, Boelle PY, Heleze E, Faye O, Loucoubar C, Magassouba N, et al. Chains of transmission and control of Ebola virus disease in Conakry, Guinea, in 2014: an observational study. Lancet Infect Dis. 2015;Jan 22(14):1–7.
15. Yamin D, Gertler S, Ndeffo-Mbah ML, Skrip LA, Fallah M, Nyenswah TG, et al. Effect of Ebola progression on transmission and control in Liberia. Ann Intern Med. 2015 Jan 6;162(1):11–7.
16. Lewnard JA, Ndeffo Mbah ML, Alfaro-Murillo JA, Altice FL, Bawo L, Nyenswah TG, et al. Dynamics and control of Ebola virus transmission in Montserrado, Liberia: a mathematical modelling analysis. Lancet Infect Dis. Oxford; 2014;14(12):1189–95.
17. Stadler T, Kühnert D, Rasmussen DA, du Plessis L. Insights into the early epidemic spread of ebola in sierra leone provided by viral sequence data. PLoS Curr. 2014;6.
18. Alizon S, Lion S, Murall CL, Abbate JL. Quantifying the epidemic spread of Ebola virus (EBOV) in Sierra Leone using phylodynamics. Virulence. 2014;5(8):825–7.
19. Volz E, Pond S. Phylodynamic analysis of ebola virus in the 2014 sierra leone epidemic. PLoS Curr. 2014;6.
20. Scarpino S V, Iamarino A, Wells C, Yamin D, Ndeffo-Mbah M, Wenzel NS, et al. Epidemiological and viral genomic sequence analysis of the 2014 ebola outbreak reveals clustered transmission. Clin Infect Dis. 2015;60(7):1079–82.
